# Identification and functional characterization of novel mutations including frameshift mutation in exon 4 of *CSF1R* in patients with adult-onset leukoencephalopathy with axonal spheroids and pigmented glia

**DOI:** 10.1007/s00415-018-9017-2

**Published:** 2018-08-22

**Authors:** Takeshi Miura, Naomi Mezaki, Takuya Konno, Akio Iwasaki, Naoyuki Hara, Masatomo Miura, Michitaka Funayama, Yuki Unai, Yuichi Tashiro, Kenji Okita, Takeshi Kihara, Nobuo Ito, Yoichi Kanatsuka, David T. Jones, Norikazu Hara, Takanobu Ishiguro, Takayoshi Tokutake, Kensaku Kasuga, Hiroaki Nozaki, Dennis W. Dickson, Osamu Onodera, Zbigniew K. Wszolek, Takeshi Ikeuchi

**Affiliations:** 1 Department of Molecular Genetics, Brain Research Institute, Niigata University, 1-757 Asahimachi, Chuo-ku, Niigata, 951-8585 Japan; 2 Department of Neurology, Brain Research Institute, Niigata University, 1-757 Asahimachi, Chuo-ku, Niigata, 951-8585 Japan; 3 Department of Neurology, Mayo Clinic, 4500 San Pablo Road, Jacksonville, FL 32224 USA; 4 Department of Neurology, Dokkyo Medical University, 880 Kitakobayashi, Mibu-machi, Shimotsuga, 321-0293 Japan; 5 Department of Clinical Neuroscience and Therapeutics, Hiroshima University Graduate School of Biomedical and Health Science, 1-2-3 Kasumi, Minami-ku, Hiroshima, 734-8553 Japan; 6 Department of Neurology, Graduate School of Medical Sciences, Kumamoto University, 1-1-1 Honjo,Chuo-ku, Kumamoto, 860-8555 Japan; 7 Department of Neuropsychiatry, Ashikaga Red Cross Hospital, 284-1 Yobe, Ashikaga, 326-0843 Japan; 8 Department of Neurology, Sumitomo Hospital, 5-3-20 Nakanoshima, Kita-ku, Osaka, 530-0005 Japan; 9 Department of Neurology, National Hospital Organization Mito Medical Center, 280 Sakuranosato, Ibarakimachi, Higashiibaraki 311-3193 Japan; 10 Department of Neurology, Nagoya City University Graduate School of Medical Sciences, Kawasumi 1-40, Mizuho-ku, Nagoya, 467-8601 Japan; 11 Department of Neurology, Rakuwakai Otowa Rehabilitation Hospital, 32-1 Koyamakitamizocho, Yamashina-ku, Kyoto, 607-8113 Japan; 12 Department of Neurology, Suzuka General Hospital, 1275-53 Yamanohana, Yasuzukacho, Suzuka, 513-8630 Japan; 13 Department of Neurology, Yokohama Municipal Citizen’s Hospital, 56 Okazawacho, Hodogaya-ku, Yokohama, 240-8555 Japan; 14 Department of Neurology, Mayo Clinic, 200 First Street S.W., Rochester, MN 55905 USA; 15 Graduate School of Health Sciences, Niigata University, 1-757 Asahimachi, Chuo-ku, Niigata, 951-8585 Japan; 16 Department of Neuroscience, Mayo Clinic, 4500 San Pablo Road, Jacksonville, FL 32224 USA

**Keywords:** ALSP, CSF1R, Leukoencephalopathy, Haploinsufficiency, HDLS

## Abstract

**Objective:**

Adult-onset leukoencephalopathy with axonal spheroids and pigmented glia (ALSP) is caused by mutations in *CSF1R*. Pathogenic mutations in exons 12–22 including coding sequence of the tyrosine kinase domain (TKD) of *CSF1R* were previously identified. We aimed to identify *CSF1R* mutations in patients who were clinically suspected of having ALSP and to determine the pathogenicity of novel *CSF1R* variants.

**Methods:**

Sixty-one patients who fulfilled the diagnostic criteria of ALSP were included in this study. Genetic analysis of *CSF1R* was performed for all the coding exons. The haploinsufficiency of CSF1R was examined for frameshift mutations by RT-PCR. Ligand-dependent autophosphorylation of CSF1R was examined in cells expressing CSF1R mutants.

**Results:**

We identified ten variants in *CSF1R* including two novel frameshift, five novel missense, and two known missense mutations as well as one known missense variant. Eight mutations were located in TKD. One frameshift mutation (p.Pro104LeufsTer8) and one missense variant (p.His362Arg) were located in the extracellular domain. RT-PCR analysis revealed that the frameshift mutation of p.Pro104LeufsTer8 caused nonsense-mediated mRNA decay. Functional assay revealed that none of the mutations within TKD showed autophosphorylation of CSF1R. The p.His362Arg variant located in the extracellular domain showed comparable autophosphorylation of CSF1R to the wild type, suggesting that this variant is not likely pathogenic.

**Conclusions:**

The detection of the *CSF1R* mutation outside of the region-encoding TKD may extend the genetic spectrum of ALSP with *CSF1R* mutations. Mutational analysis of all the coding exons of *CSF1R* should be considered for patients clinically suspected of having ALSP.

**Electronic supplementary material:**

The online version of this article (10.1007/s00415-018-9017-2) contains supplementary material, which is available to authorized users.

## Introduction

Adult-onset leukoencephalopathy with axonal spheroids and pigmented glia (ALSP) is an autosomal dominant neurological disorder that predominantly affects the cerebral white matter [1]. ALSP encompasses two similar entities previously known as hereditary diffuse leukoencephalopathy with spheroids (HDLS) and pigmentary orthochromatic leukodystrophy (POLD) [1]. ALSP is clinically characterized by executive dysfunction, memory decline, personality changes, motor impairments, and seizures [2]. Frontal lobe syndrome in ALSP is characterized by loss of judgment, lack of social inhibitors, lack of insight, and motor persistence, which usually appears early in the disease course. The mean age at onset is 43 years ranging from 18 to 78 years [3]. Patients with ALSP eventually become bedridden with a mean disease duration of 6.8 years from onset to death [3].

Mutation in the colony stimulating factor 1 receptor (*CSF1R*) was identified as the cause of ALSP [4]. To date, 58 pathological mutations in *CSF1R* have been identified [3]. All reported mutations were found in exons 12−22 including the coding sequence of the tyrosine kinase domain (TKD) of *CSF1R* [1–3]. We previously reported that *CSF1R* mutation-mediated pathogenesis may be explained by haploinsufficiency or the loss of CSF1R-mediated signals [5].

In this study, we attempted to identify the *CSF1R* mutations in patients who were referred to our institute for genetic analysis. By this analysis, we found novel and previously reported *CSF1R* mutations including a novel frameshift in exon 4 outside of TKD and examined the pathogenicity of novel *CSF1R* variants.

## Methods

### Patients

Sixty-one patients from 59 pedigrees who fulfilled the diagnostic criteria of probable or possible ALSP were included in this study [6]. All the patients or their relatives provided their informed consent. This study was approved by the institutional review board committees of Niigata University and Mayo Clinic Florida.

### Genetic and mRNA analyses

Genomic DNA and total RNA were isolated from peripheral leukocytes using standard methods. Mutational analysis of *CSF1R* was performed using sequences of both strands of all PCR-amplified coding exons and flanking intronic sequences as previously described [4, 5]. Briefly, all the exons and exon–intron boundaries of *CSF1R* were amplified by PCR, followed by Sanger sequence. We conducted in silico analysis using the PolyPhen-2, SIFT, PROVEAN, VEP and CADD algorithms to predict the pathogenicity of novel missense or indel mutations. Total RNA was extracted from peripheral leukocytes. Complementary DNA was synthesized using ReverTra Ace^®^ (TOYOBO, Osaka, Japan).

### Functional and immunoblot analyses

Mutagenesis was performed to generate cDNA of mutant *CSF1R* as previously described [5]. HEK293T cells were transiently transfected with wild-type or mutant CSF1R, and were cultured in a medium containing 10% fetal bovine serum (FBS). In another set of experiments, HEK293T cells were stimulated with the ligands of CSF1R, CSF1, and IL-34, in the absence of FBS to induce autophosphorylation of CSF1R. Detergent cell lysates were subjected to immunoblot analysis using antibodies including C-20 (C-terminus of CSF1R, Santa Cruz Biotechnology, Dallas, TX, USA) and B-8 (N-Terminus of CSF1R, Santa Cruz Biotechnology, Dallas, TX, USA) to detect total CSF1R. CSF1R phosphorylated at Tyr546, Tyr708, and Tyr723 was detected using specific anti-phosphorylated CSF1R antibodies (Cell Signaling Technology, Beverly, MA, USA).

### Clinical and neuroimaging analyses

Clinical presentations and neuroimaging findings of the patients carrying *CSF1R* variants were retrospectively evaluated. MRI was conducted for routine diagnostic purposes using 1.5T systems in all the patients (*n* = 11). CT using thin slices was performed in seven patients.

### Neuropathological examination

Neuropathological examination of brain samples from patient 10 was performed as previously described [7]. Briefly, tissue sections were embedded in paraffin, and 5-µm-thick sections were mounted on glass slides for histopathological and immunohistochemical analyses. The paraffin-embedded sections were stained with hematoxylin and eosin (H&E) and Luxol fast blue. Sections were also stained with antibodies against CD68, phosphorylated neurofilaments, and αB-crystallin.

### Statistical analysis

The signal intensity of immunoblots was semiquantitatively analyzed using a LAS 4000 analyzer (GE Health Science, Piscataway, NJ, USA). The intensity of each autophosphorylation signal of CSF1R was normalized to the total amount of CSF1R. All statistical tests were run in GraphPad Prism 5 (GraphPad Software, La Jolla, CA, USA). Data are presented as mean ± SEM. Statistical analysis was performed by two-way ANOVA with Bonferroni correction.

## Results

### Identification of *CSF1R* mutations

**Fig. 1:**
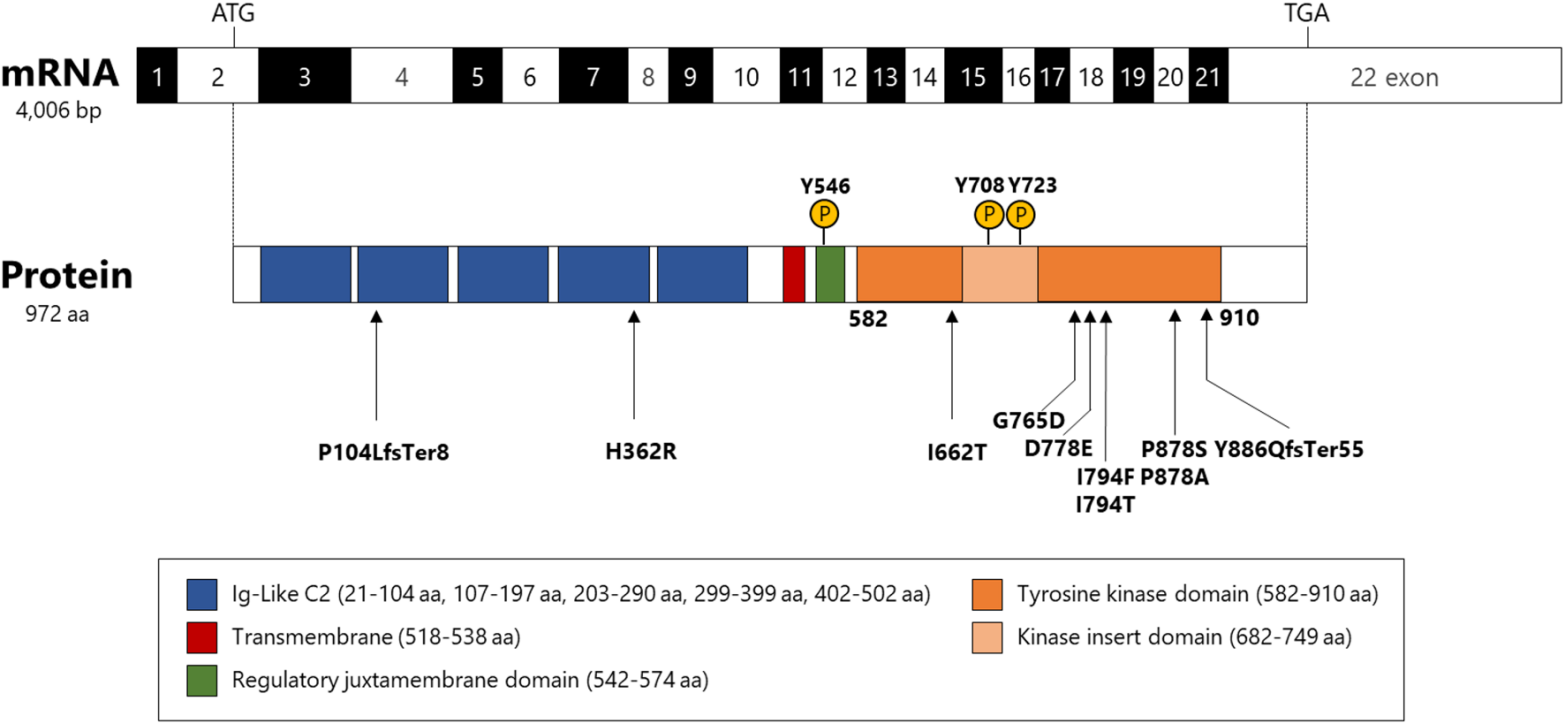
Illustration of *CSF1R* structure and location of mutations. The positions of the variants identified in this study are indicated by arrows. The phospho-specific antibodies used in this study against CSF1R are also depicted. The position of each domain is shown on the basis of information from UniProt (http://www.uniprot.org/uniprot/P07333)

Eight missense variants including five novel mutations (p.Ile662Thr, p.Asp778Glu, p.Ile794Phe, p.Pro878Ser, and p.Pro878Ala), two known missense mutations (p.Gly765Asp and p.Ile794Thr), one known homozygote variant (p.His362Arg), and two frameshift mutations (p.Pro104LeufsTer8 and p.Tyr886GlnfsTer55) in *CSF1R* were identified (Fig. 1). Eight mutations were located in TKD. One frameshift mutation (p.Pro104LeufsTer8) was located in the extracellular domain and was predicted to cause nonsense-mediated RNA decay (NMD) [8]. Results of in silico analysis of *CSF1R* variants in ALSP patients revealed that these mutations within TKD are predicted to be pathogenic with high probability (Electronic Supplementary Table 1). Furthermore, these mutations were not found in the ExAC database, supporting the pathogenicity of these mutations, except for p.Ile794Thr, which is the most frequently reported *CSF1R* mutation (Electronic Supplementary Table 1). The p.His362Arg variant located outside TKD appeared to be nonpathogenic, because the allele frequency of p.His362Arg was 0.049 in the ExAC database.

### Analysis of mRNA expression of frameshift *CSF1R* mutations

**Fig. 2:**
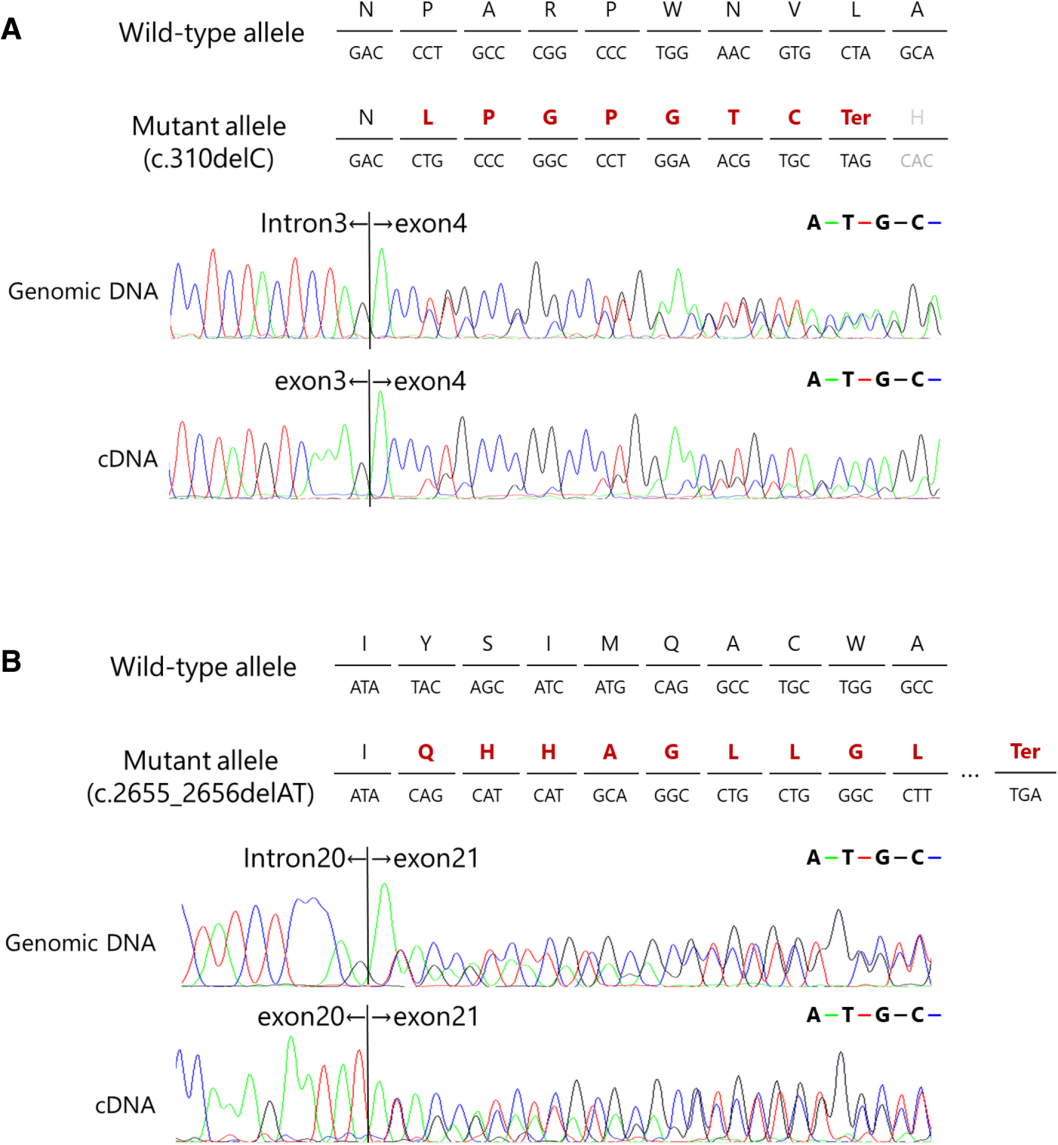
mRNA expression of frameshift *CSF1R* mutations. **a** Frameshift mutation in exon 4 (p.Pro104LeufsTer8), which was predicted to undergo NMD. The expression level of the mutant allele was markedly decreased, suggesting that this frameshift mutation results in NMD of mutant mRNA. **b** Frameshift mutation in exon 21 (p.Tyr886GlnfsTer55), which was predicted not to undergo NMD because the premature terminal codon is generated in exon 22, the last exon of *CSF1R*. The expression level of the mutant allele was comparable to that of the wild-type allele

We performed reverse transcription (RT)-PCR analysis using mRNA obtained from peripheral leukocytes to examine the mRNA expression of the frameshift mutations of p.Pro104LeufsTer8 and p.Tyr886GlnfsTer55. The analysis revealed that the expression level of the mutant allele derived from the p.Pro104LeufsTer8 mutation was markedly lower than that of the normal allele (Fig. 2a). This finding suggests that the p.Pro104LeufsTer8 frameshift undergoes NMD. On the other hand, the mutant allele derived from the p.Tyr886GlnfsTer55 frameshift mutation was expressed at a level comparable to that of the wild-type allele (Fig. 2b).

### Defective autophosphorylation of mutant CSF1R

**Fig. 3:**
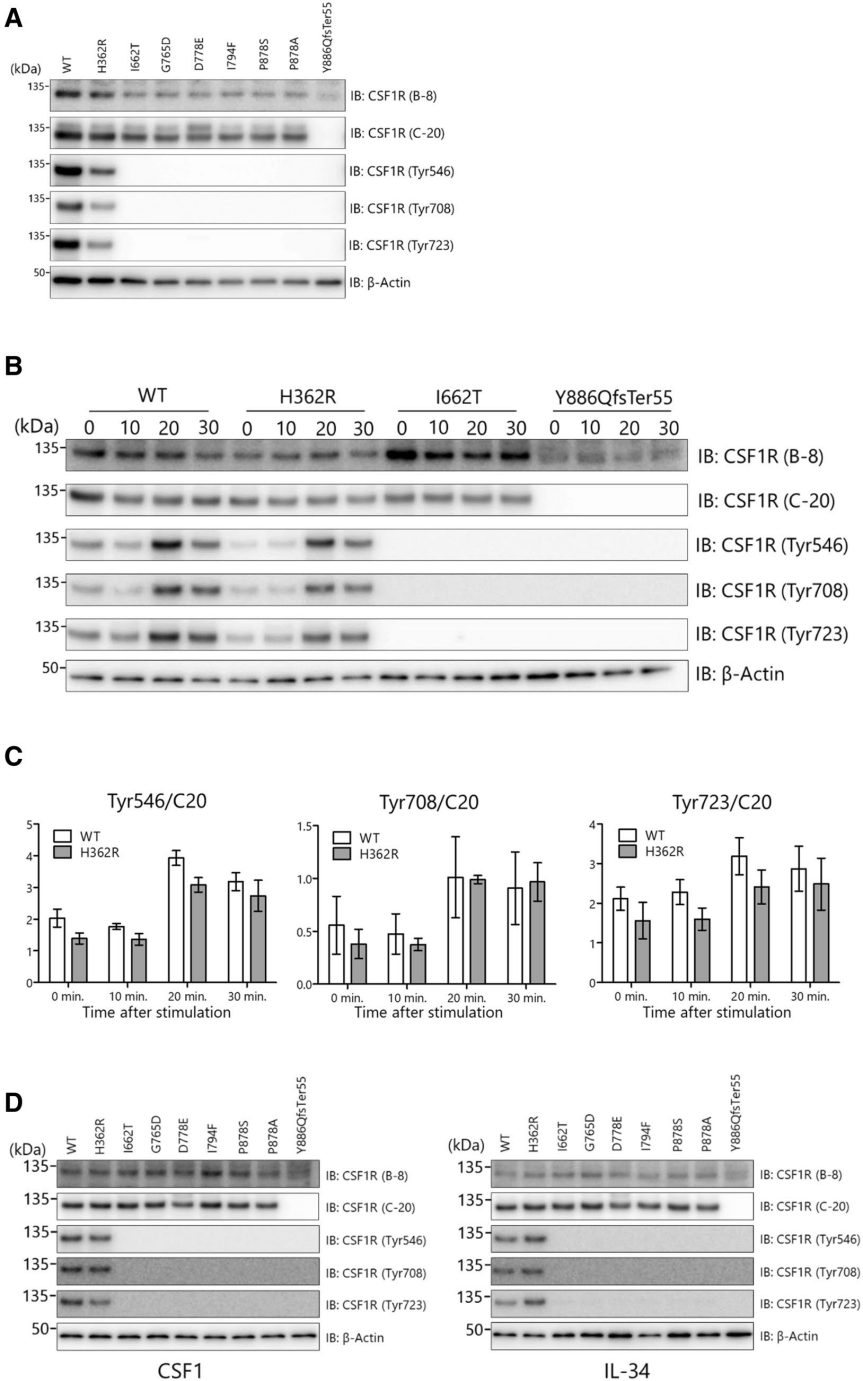
Functional assay of mutant CSF1Rs. **a** HEK293T cells transfected with the wild type or mutant CSF1R were cultured in a medium containing 10% FBS. Western blot analysis using anti-CSF1R antibodies (B-8 and C-20) revealed comparable expression levels of total CSF1R between the wild-type and mutant CSF1Rs. Note that mutant CSF1R with Tyr886GlnfsTer55 lacking the C-terminal portion of CSF1R showed no band reactive to the anti-C-terminal antibody (C-20), but was recognized as a slightly smaller molecular weight by the anti-N-terminal antibody (B-8). None of the variants within TKD revealed autophosphorylation of CSF1R. The p.His362Arg variant showed a comparable degree of autophosphorylation of CSF1R to the wild type. The anti-β-actin antibody was used as a loading control. **b** Ligand-dependent autophosphorylation of CSF1R was examined in cells transiently transfected with the wild type or variant CSF1Rs. Detergent-extracted lysates were collected at the indicated time (min) after CSF1 stimulation. Increased levels of phosphorylated CSF1R were observed to be comparable in cells expressing the wild type and p.His362Arg. In contrast, neither of the mutants within TKD underwent autophosphorylation of CSF1R following CSF1 stimulation. **c** Results of semiquantification of immunoblot data (*n* = 3). There is no statistically significant difference in the degree of ligand-dependent autophosphorylation between the wild type and the p.His362Arg variant. Data are presented as mean ± SEM. **d** Ligand-dependent autophosphorylation of CSF1R. Cells were stimulated with CSF1 (left panel) or IL-34 (right panel). Detergent-extracted lysates were collected 20 min after ligand stimulation. Phosphorylated CSF1R levels were comparable between cells expressing wild-type CSF1R and p.His362Arg, whereas cells expressing mutant CSF1Rs within TKD showed no autophosphorylation

To examine the pathogenicity of missense variants, we examined the autophosphorylation of CSF1R in HEK293T cells expressing wild-type or mutant CSF1R. CSF1R phosphorylated at Tyr546, Tyr708, and Tyr723 was detected in cells expressing wild-type CSF1R cultured in the presence of FBS (Fig. 3a). No autophosphorylation of CSF1R was observed in cells expressing missense variants including p.Ile662Thr, p.Gly765Asp, p.Asp778Glu, p.Ile794Phe, p.Pro878Ser, or p.Pro878Ala in TKD, and p.Tyr886GlnfsTer55 (Fig. 3a). The p.His362Arg variant located in the extracellular domain showed a comparable degree of autophosphorylation of CSF1R to the wild type (Fig. 3a).

In another set of experiments, ligand-induced autophosphorylation of CSF1R was examined. None of the missense mutations within TKD or the frameshift mutation p.Tyr886GlnfsTer55 showed autophosphorylation upon the stimulation of CSF1 or IL-34 (Fig. 3b, d). The p.His362Arg variant showed a comparable degree of autophosphorylation of CSF1R to the wild type, suggesting that this variant does not cause the loss of CSF1R-mediated signals (Fig. 3b, c, d).

### Clinical and neuroimaging characteristics

The clinical characteristics of 11 patients are shown in Table 1. Familial occurrence was observed in three patients. The case of a sibling of patient 4 with the p.Gly765Asp mutation was previously reported [5]. Eight patients apparently had no family history of the disease. The mean age at onset of the patients was 42.8 years ranging from 22 to 60 years. Dementia was observed in all the patients, and pyramidal signs were observed in 6 (55%) of the 11 patients.

**Table 1:** Clinical characteristics of patients with *CSF1R* variants

Patient no.	Variant	Sex	Family history	Age at onset (year)	Age at exam (year)	Initial symptoms	Dementia	Pyramidal signs	Parkinsonism	Seizure	Diagnostic criteria [6]
1	p.Pro104LeufsTer8 c.310delC	F	−	22	24	Right hemiparesis	+	+	−	−	Possible
2	p.His362Arg (homo) c.1085A>G	M	−	25	29	Amnesia	+	−	+	−	Possible
3	p.Ile662Th c.1985T>C	M	+	40	46	Amnesia	+	+	−	−	Probable
4	p.Gly765Asp c.2294G>A	M	+	44	47	Bradykinesia	+	−	+	−	Possible
5	p.Asp778Glu c.2334C>A	F	−	60	66	Limb apraxia	+	−	−	−	Possible
6	p.Ile794Phe c.2380A>T	M	−	56	57	Difficulty in concentration	+	+	+	−	Possible
7	p.Ile794Thr c.2381T>C	F	−	33	36	Weakness in the right hand	+	−	−	−	Probable
8	p.Ile794Thr c.2381T>C	M	−	43	56	Apathy	+	+	−	+	Probable
9	p.Pro878Ser c.2632C>T	M	−	45	55	Cognitive impairment	+	+	−	+	Probable
10	p.Pro878Ala c.2632C>G	M	+	57	62	Depression, anxiety	+	+	+	−	Probable
11	p.Tyr886GlnfsTer55 c.2655_2656delAT	M	−	46	46	Disorientation	+	−	−	−	Possible

**Fig. 4:**
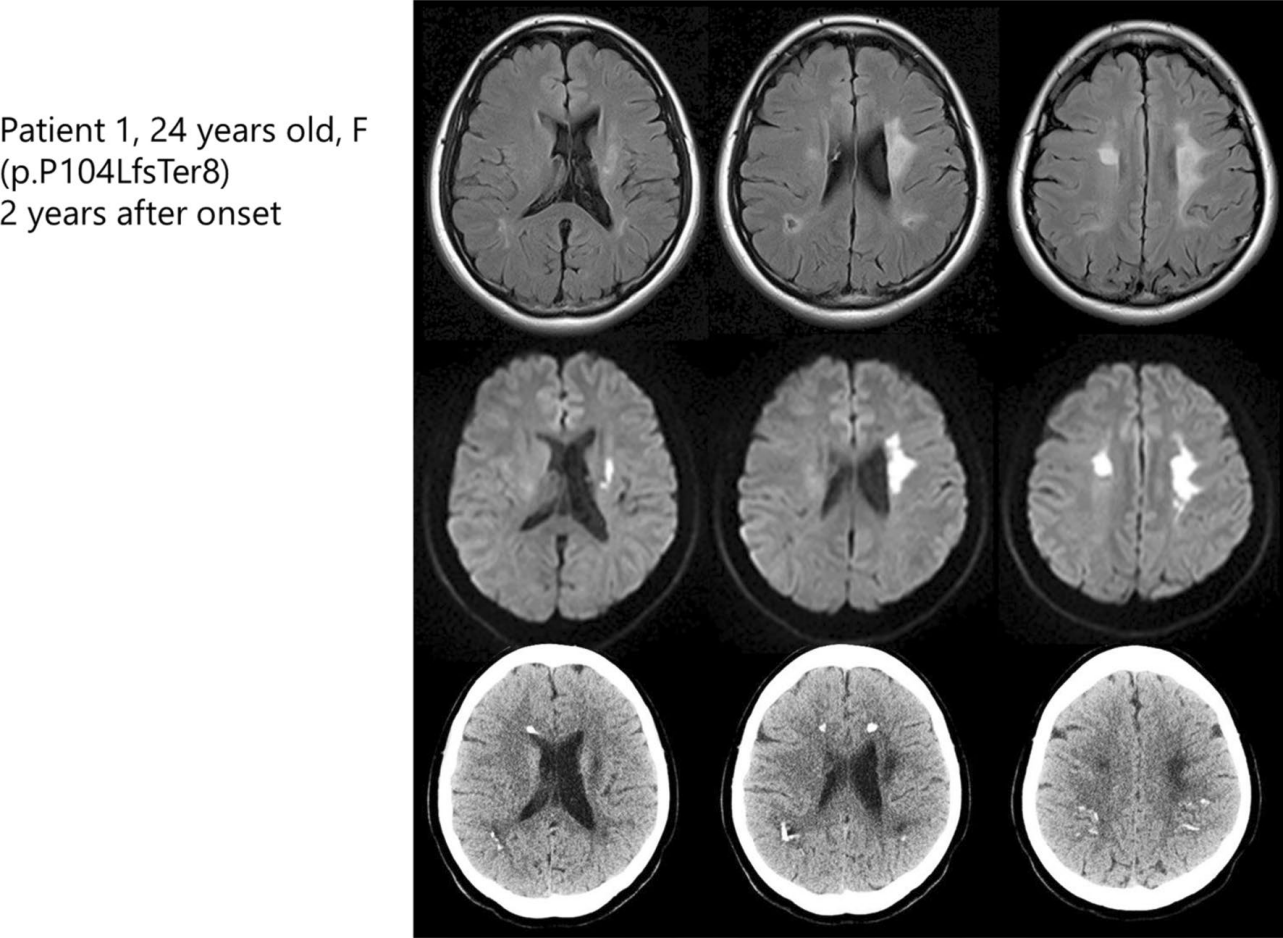
Brain MRI and CT findings of ALSP patient with the frameshift mutation in exon 4. Characteristic hyperintensities in the white matter are observed in fluid-attenuated inversion recovery MR images (upper panels). Diffusion-weighted MR images revealed high-intensity lesions with left-side predominance (middle panels). CT scan revealed small spotty calcifications in the frontal and parietal white matter (lower panels)

Neuroimaging features are shown in Electronic Supplementary Table 2. The white matter lesions detected by MRI were usually distributed bilaterally, but could be distributed asymmetrically (Fig. 4, Electronic Supplementary Fig. 1). Hyperintensity lesions on diffusion-weighted MR images were observed in 8 of the 11 patients (73%). Calcifications were observed in six (86%) of the seven patients by CT (Electronic Supplementary Table 2, Electronic Supplementary Fig. 2).

### Neuropathological findings

Patient 10 was a 64-year-old man from family 6510 and his depression and anxiety insidiously developed at the age of 57. Subsequently, he exhibited cognitive decline and personality/behavioral changes. At the age of 62 years, he developed parkinsonism characterized by rigidity, bradykinesia, and gait disturbance. He became totally dependent by the age of 63 years and died at 64.

**Fig. 5:**
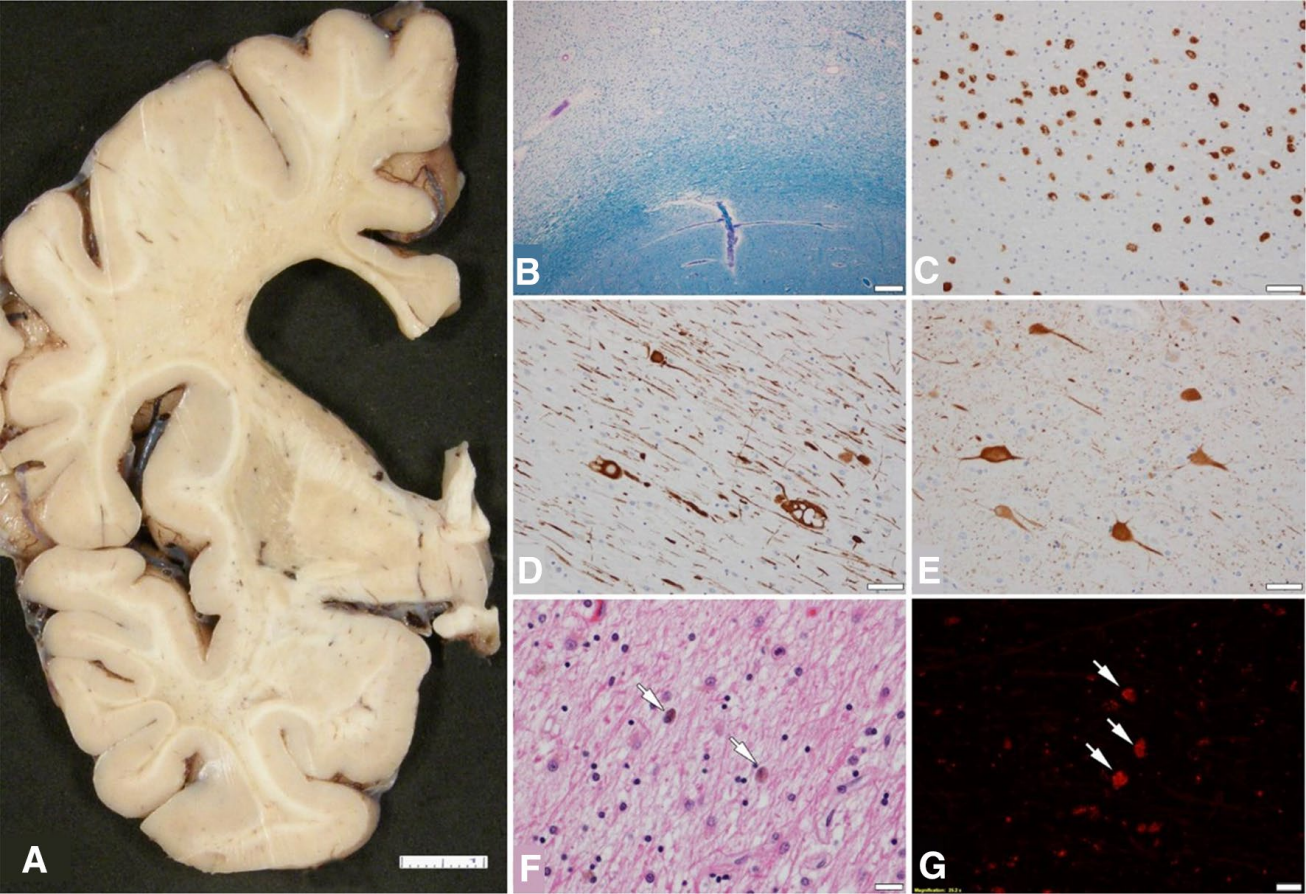
Neuropathological findings of ALSP patient with p.Pro878Ala mutation. **a** Coronal section of cerebrum showing frontal white matter pathology. **b** Frontal white matter myelin loss on Luxol fast blue staining (note the relative sparing of subcortical arcuate fibers). **c** Macrophages in frontal white matter (CD68 immunohistochemistry). **d** Axonal spheroids in frontal white matter (phosphorylated neurofilament immunohistochemistry). **e** Ballooned neurons in frontal cortex (αB-crystallin immunohistochemistry). **f** Macrophages in white matter with brown granular pigment (arrows) (H&E stain). **g** Pigment in macrophages in white matter showing autofluorescence. Bar in **a** = 1 cm; bar in **b** = 200 µm; bars in **c, d**, and **e** = 50 µm; bars in **f** and **g** = 20 µm

The autopsy examination revealed marked frontal white matter pathology with enlargement of the frontal horn of the lateral ventricle and thinning of the corpus callosum as the gross findings (Fig. 5a). The cerebral white matter showed myelin loss on Luxol fast blue staining (Fig. 5b). There were pigment-laden macrophages visible on H&E staining and the pigment was autofluorescent (Fig. 5c, f, g). Immunohistochemical analysis using an anti-phosphorylated neurofilament antibody revealed axonal spheroids (Fig. 5d). Numerous ballooned neurons were detected in the frontal cortex (Fig. 5e). These neuropathological findings were consistent with ALSP [7].

## Discussion

In this study, we identified seven novel and two previously reported mutations in *CSF1R* among patients who fulfilled the diagnostic criteria of ALSP [6]. This study provides several noteworthy insights relevant to the role of *CSF1R* mutations in ALSP.

First, we identified a novel frameshift mutation caused by single-nucleotide deletion (c.310delC) in exon 4 resulting in p.Pro104LeufsTer8 located outside of TKD. All the previously reported mutations in ALSP were found within exons 12−22 including the coding sequence of TKD [3–5]. The frameshift mutation p.Pro104LeufsTer8 generates a premature stop codon, which was predicted to cause NMD [8]. Our expression analysis revealed that the level of mRNA expression derived from the mutant allele was substantially decreased as predicted (Fig. 2a). These findings suggest that the p.Pro104LeufsTer8 mutation causes ALSP owing to the haploinsufficiency of *CSF1R*.

Second, we identified another novel frameshift mutation caused by two-nucleotide deletion (c.2655_2656delAT) in exon 21 resulting in p.Tyr886GlnfsTer55. This frameshift mutation does not fulfill the criterion of NMD because the premature stop codon is generated within exon 22, the last exon of *CSF1R*. Our expression analysis revealed that the mRNA expression level of the mutant allele was comparable to that of the wild-type allele (Fig. 2b). These findings suggest that this frameshift mutation does not cause *CSF1R* haploinsufficiency. Because this mutation is located within TKD, we examined whether the mutant CSF1R of p.Tyr886GlnfsTer55 affects autophosphorylation upon ligand stimulation. Our examination revealed that the mutant CSF1R of p.Tyr886GlnfsTer55 did not show autophosphorylation (Fig. 3a, b, d). Thus, the pathogenic mechanism caused by p.Tyr886GlnfsTer55 mutation appears to be the loss of CSF1R-mediated signals.

Third, we showed that the autophosphorylation of novel missense variants (p.Ile662Thr, p.Asp778Glu, p.Ile794Phe, p.Pro878Ser, and p.Pro878Ala) within TKD was impaired. These findings suggest that these mutants within TKD are the causal factors [4, 5]. As previously reported [3], pathogenic mutations identified in this study occur more frequently in the distal part of TKD than in the proximal part. There is apparently no significant difference in the degree of autophosphorylation impairment between the p.Ile662Thr mutant within the proximal part of TKD and other mutants within the distal part of TKD.

Finally, we identified the homozygous *CSF1R* variant of p.His362Arg located in the extracellular domain in patient 2 who fulfilled the possible criteria of ALSP. We considered that the p.His362Arg missense variant is not the causative factor for the following reasons. First, the degree of autophosphorylation of this variant was comparable to that of the wild type. Second, this variant was reported in the ExAC database to have a frequency of 0.049 in the general population and 0.368 in the East Asian population. Thus, the cause of leukoencephalopathy in this patient is not likely to be this *CSF1R* variant. Recently, mutations in *AARS2* have been reported in ALSP patients who lack *CSF1R* mutations [9]. We performed genetic analysis of *AARS2* and found no causative mutation in this patient.

The clinical and neuropathological findings with *CSF1R* mutations in this study were consistent with those reported previously [1–3, 7]. Indeed, all the patients in this study fulfilled possible or probable diagnostic criteria for ALSP [6]. The MRI findings of the patients were similar to those reported previously (Supplementary Table 2) [10]. Attention should be paid to patient 1 in whom signal changes detected by MRI were observed with marked left-side predominance (Fig. 4). Brain calcifications were observed in all the patients with *CSF1R* mutation, supporting the diagnostic value of this finding for ALSP [11].

In conclusion, the detection of the *CSF1R* mutation outside of exons 12−22 may extend the genetic spectrum of ALSP with *CSF1R* mutations. Mutational analysis of all the exons of *CSF1R* should be considered for patients clinically suspected of having ALSP.

## Electronic supplementary material

Below is the link to the electronic supplementary material.

Supplementary material 1 (PDF 25 KB)

Supplementary material 2 (PDF 865 KB)

## References

[1] Nicholson AM, Baker MC, Finch NA (2013). *CSF1R* mutations link POLD and HDLS as a single disease entity. Neurology.

[2] Lynch DS, Jaunmuktane Z, Sheerin UM (2016). Hereditary leukoencephalopathy with axonal spheroids: a spectrum of phenotypes from CNS vasculitis to parkinsonism in an adult onset leukodystrophy series. J Neurol Neurosurg Psychiatry.

[3] Konno T, Yoshida K, Mizuno T (2017). Clinical and genetic characterization of adult-onset leukoencephalopathy with axonal spheroids and pigmented glia associated with *CSF1R* mutation. Eur J Neurol.

[4] Rademakers R, Baker M, Nicholson AM (2012). Mutations in the *colony stimulating factor 1 receptor* (*CSF1R*) gene cause hereditary diffuse leukoencephalopathy with spheroids. Nat Genet.

[5] Konno T, Tada M, Tada M (2014). Haploinsufficiency of *CSF-1R* and clinicopathologic characterization in patients with HDLS. Neurology.

[6] Konno T, Yoshida K, Mizuta I (2018). Diagnostic criteria for adult-onset leukoencephalopathy with axonal spheroids and pigmented glia due to *CSF1R* mutation. Eur J Neurol.

[7] Baba Y, Ghetti B, Baker MC (2006). Hereditary diffuse leukoencephalopathy with spheroids: clinical, pathologic and genetic studies of a new kindred. Acta Neuropahol.

[8] Kuzmiak HA, Maquat LE (2006). Applying nonsense-mediated mRNA decay research to the clinic: progress and chalenges. Trends Mol Med.

[9] Lynch DS, Zhang WJ, Lakshmanan R (2016). Analysis of mutations in *AARS2* in a series of CSF1R-negative patients with adult-onset leukoencephalopathy with axonal spheroids and pigmented glia. JAMA Neurol.

[10] Sundal C, Van Gerpen JA, Nicholson AM (2012). MRI characteristics and scoring in HDLS due to CSF1R gene mutations. Neurology.

[11] Konno T, Broderick DF, Mezaki N (2017). Diagnostic value of brain calcifications in adult-onset leukoencephalopathy with axonal spheroids and pigmented glia. AJNR Am J Neuroradiol.

