# Supplementary material for: Identification and functional characterization of novel mutations including frameshift mutation in exon 4 of CSF1R in patients with adult-onset leukoencephalopathy with axonal spheroids and pigmented glia
Source: J Neurol. 2018 Aug 22;265(10):2415–24. doi: 10.1007/s00415-018-9017-2 (PMC6182692; doi:10.1007/s00415-018-9017-2)
Supplement: Supplementary file 1 — Supplementary material 1 (PDF 25 KB) [file 415_2018_9017_MOESM1_ESM.pdf]

Supplementary Table 1 *In silico* analysis of variants in this study

Patient No.	Variant	Exon	domain	rs	ExAC allele frequency	*In silico* analysis				
						Polyphen-2	SIFT	PROVEAN	VEP IMPACT	CADD score
1	p.Pro104LeufsTer8 c.310delC	4	Ig-like C2	Novel	-	-	-	-	HIGH	25.20
2	p.His362Arg (homo) c.1085A>G	8	Ig-like C2	rs10079250	0.049 (East Asia 0.364)	0.065 BENIGN	0.061 Tolerated	-3.36 Deleterious	MODERATE	10.33
3	p.Ile662Thr c.1985T>C	15	TKD (Proximal)	Novel	-	1.000 PROBABLY DAMAGING	0.001 Damaging	-4.28 Deleterious	MODERATE	27.50
4	p.Gly765Asp c.2294G>A	18	TKD (Distal)	rs690016566	-	1.000 PROBABLY DAMAGING	0.000 Damaging	-5.99 Deleterious	MODERATE	33.00
5	p.Asp778Glu c.2334C>A	18	TKD (Distal)	Novel	-	1.000 PROBABLY DAMAGING	0.000 Damaging	-3.33 Deleterious	MODERATE	23.40
6	p.Ile794Phe c.2380A>T	18	TKD (Distal)	Novel	-	1.000 PROBABLY DAMAGING	0.000 Damaging	-3.33 Deleterious	MODERATE	32.00
7, 8	p.Ile794Thr c.2381T>C	18	TKD (Distal)	rs281860274	8.95351e-06 (East Asia 0.000)	1.000 PROBABLY DAMAGING	0.000 Damaging	-4.17 Deleterious	MODERATE	28.10
9	p.Pro878Ser c.2632C>T	20	TKD (Distal)	Novel	-	1.000 PROBABLY DAMAGING	0.021 Damaging	-6.44 Deleterious	MODERATE	30.00
10	p.Pro878Ala c.2632C>G	20	TKD (Distal)	Novel	-	1.000 PROBABLY DAMAGING	0.010 Damaging	-6.44 Deleterious	MODERATE	26.60
11	p.Tyr886GlnfsTer55 c.2655_2656delAT	21	TKD (Distal)	Novel	-	-	-	-	HIGH	35.00

Polyphen-2, http://genetics.bwh.harvard.edu/pph2/; SIFT, http://sift.jcvi.org/; PROVEAN, http://provean.jcvi.org/index.php; VEP, https://asia.ensembl.org/info/docs/tools/vep/index.html; CADD score, http://cadd.gs.washington.edu/score

Supplementary Table 2. Imaging features of patients with *CSF1R* variants

Patient No.	Variant	Bilateral cerebral white matter lesion	Thinning of Corpus callosum	DWI hyper intensity	Calcification on thin slice CT	
					Frontal	Parietal
1	p.Pro104LeufsTer8<br>c.310delC	+	-	+	+	+
2	p.His362Arg (homo)<br>c.1085A>G	+	-	-	-	-
3	p.Ile662Thr<br>c.1985T>C	+	+	+	n/a	n/a
4	p.Gly765Asp<br>c.2294G>A	+	+	+	+	-
5	p.Asp778Glu<br>c.2334C>A	+	+	-	+	+
6	p.Ile794Phe<br>c.2380A>T	+	-	+	n/a	n/a
7	p.Ile794Thr<br>c.2381T>C	+	-	+	+	-
8	p.Ile794Thr<br>c.2381T>C	+	+	+	n/a	n/a
9	p.Pro878Ser<br>c.2632C>T	+	+	+	+	-
10	p.Pro878Ala<br>c.2632C>G	+	+	-	n/a	n/a
11	p.Tyr886GlnfsTer55<br>c.2655_2656delAT	+	+	+	+	-
