# Supplementary material for: Identification and functional characterization of novel mutations including frameshift mutation in exon 4 of CSF1R in patients with adult-onset leukoencephalopathy with axonal spheroids and pigmented glia
Source: J Neurol. 2018 Aug 22;265(10):2415–24. doi: 10.1007/s00415-018-9017-2 (PMC6182692; doi:10.1007/s00415-018-9017-2)
Supplement: Supplementary file 2 — Supplementary material 2 (PDF 865 KB) [file 415_2018_9017_MOESM2_ESM.pdf]

Supplementary Figure 1: MRI images of patients with *CSF1R* variants

Patient 2:
p.His362Arg,
(Homozygote)
29 years old, male
4 years after onset

Patient 3:
p.Ile662Thr
46 years old, male
6 years after onset

Patient 4:
p.Ggy765Asp
47 years old, male
3 years after onset

Patient 5:
p.Asp778Glu
66 years old, female
6 years after onset

Patient 6:
p.Ile794Phe
57 years old, male
1 year after onset

Patient 9:
p.Pro878Ser
55 years old, male
6 years after onset

Patient 10:
p.Pro878Ala
62 years old, male
5 years after onset

Patient 11:
p.Tyr886GlnfsTer55
46 years old, male
1 year after onset

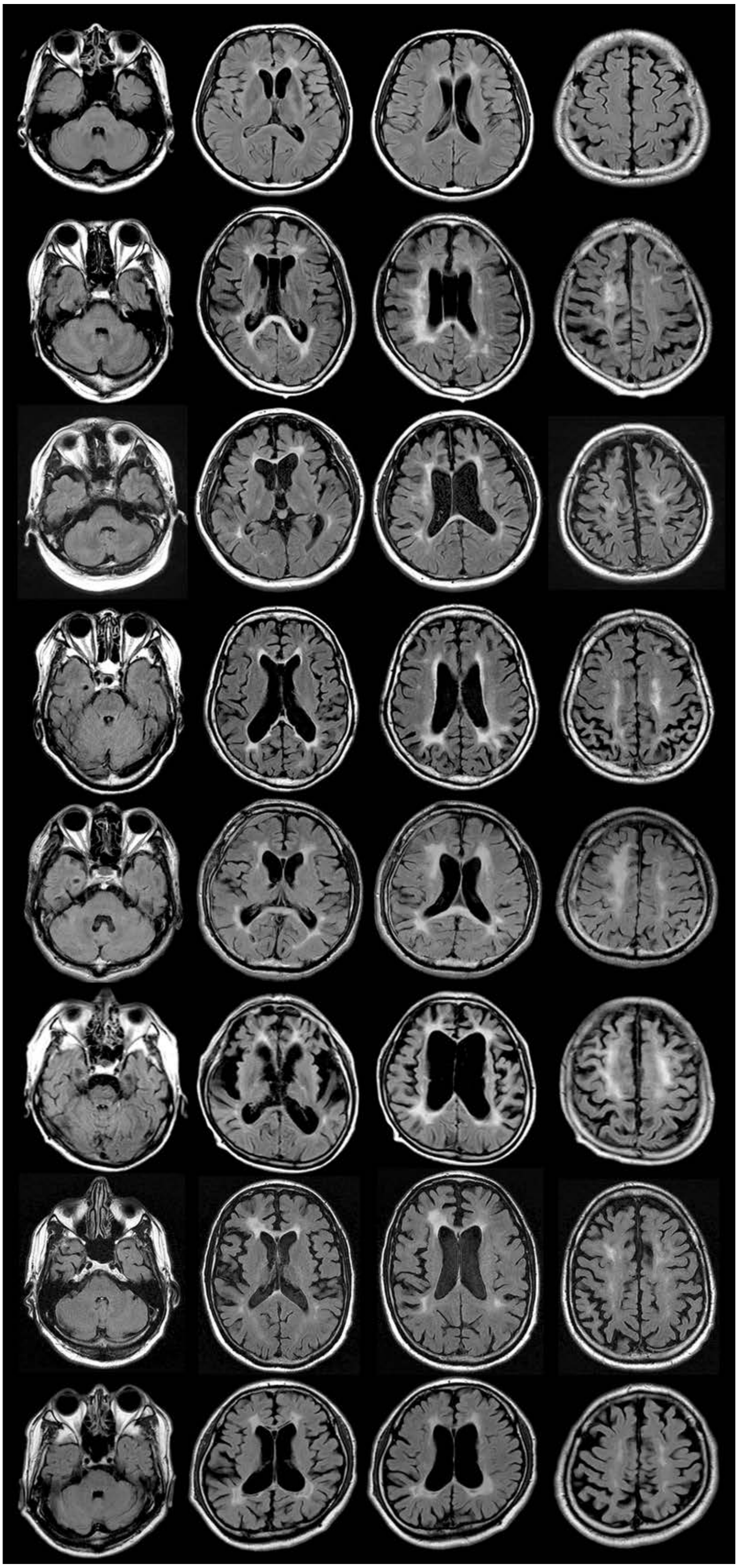

Supplementary Figure 2: CT scans of patients with *CSF1R* variants

Patient 2:
p.His362Arg
(Homozygote)
29 years old, male
4 years after onset

Patient 4:
p.Gly765Asp
47 years old, male
3 years after onset

Patient 5:
p.Asp778Glu
66 years old, female
6 years after onset

Patient 7:
p.Ile794Thr
35 years old, female
2 years after onset

Patient 9:
p.Pro878Ser
55 years old, male
6 years after onset

Patient 11:
p.Tyr886GlnfsTer55
46 years old, male
1 year after onset

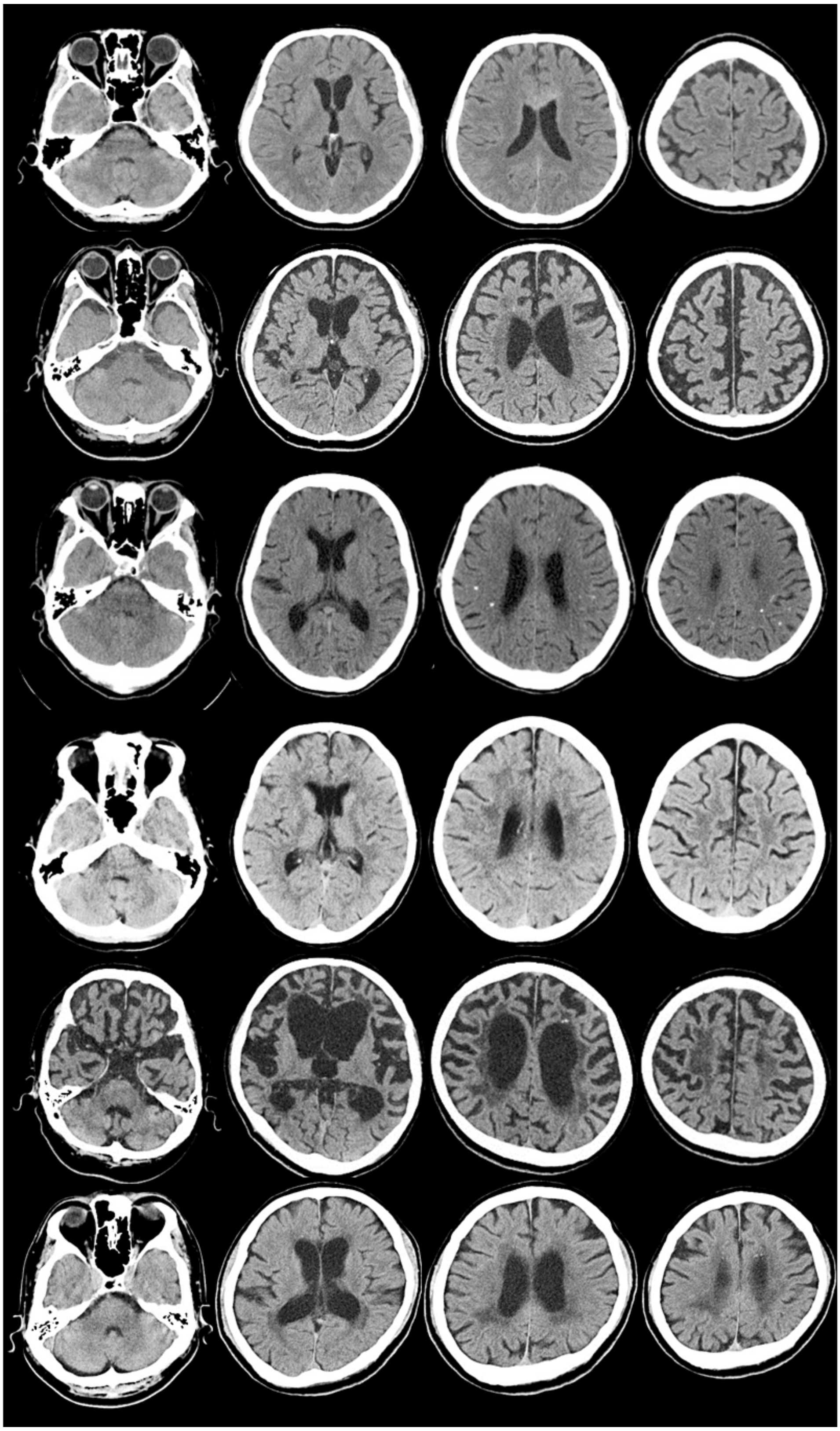

Legend:
Calcification was present in *patient 4, 5, 7, 9,* and *11*. *Patient 2* did not show calcification by CT with thin slice.
